# Immunoglobulins as steroid sparing agents in chronic relapsing inflammatory optic neuropathy

**DOI:** 10.4103/0972-2327.64629

**Published:** 2010

**Authors:** Luis I. Gonzalez-Granado

**Affiliations:** Immunodeficiencies Unit, Hospital 12 octubre, Carretera Andalucia km 5,400, P.O. 28041, Madrid, Spain

Sir,

I read with great interest the case reported by Drs Saini and Khurana titled "Chronic relapsing inflammatory optic neuropathy".[1] I do really appreciate their contribution to the knowledge of chronic relapsing inflammatory optic neuropathy. However, after reading the case I have one main concern: long-term management. It is well known that steroids can prevent reactivation of chronic relapsing inflammatory optic neuropathy. Nonetheless, we should be aware of the severe adverse effects that long-term steroids can induce in these patients. Therefore, there is an increasing interest in the use of intravenous immunoglobulins as steroid-sparing agents, as they have been demonstrated to be effective in the treatment of idiopathic inflammatory neuropathies including Guillain-Barré syndrome, chronic inflammatory demyelinating neuropathy and multifocal motor neuropathy (MMN; in this case represents the gold standard for treatment).[2,3] In the past, other steroid-sparing agents have been used with mild to severe side effects (methotrexate, cyclophosphamide, azathioprine – the most used drug within this group – and mycophenylate).[4,5] The authors could argue that deflazacort was administered on alternate days. Nevertheless, the anti-inflammatory effect is 30% higher compared with prednisolone and a better risk/benefit ratio has only been demonstrated for the bone-sparing effect.[6]

This is why steroid sparing agents should be strongly considered (especially i.v. immunoglobulins) for the management of this condition.
